# Supplementary material for: A Critical View of the Application of the APEX Software (Aqueous Photochemistry of Environmentally-Occurring Xenobiotics) to Predict Photoreaction Kinetics in Surface Freshwaters
Source: Molecules. 2019 Dec 18;25(1):9. doi: 10.3390/molecules25010009 (PMC7017383; doi:10.3390/molecules25010009)
Supplement: Supplementary file 1 [file molecules-25-00009-s001.zip › 3.2.4_gcc-4.4.0.zip!/3.2.4_gcc-4.4.0/3.2.4_gcc-4.4.0/doc/octave/html/interpreter/Input-Parameter-Checking-in-Oct_002dFiles.html]

Input Parameter Checking in Oct-Files - Untitled


Next: Exception and Error Handling in Oct-Files,
Previous: Allocating Local Memory in Oct-Files,
Up: Oct-Files


---

#### A.1.11 Input Parameter Checking in Oct-Files

As oct-files are compiled functions they have the possibility of causing
Octave to abort abnormally. It is therefore important that
each and every function has the minimum of parameter
checking needed to ensure that Octave behaves well.

The minimum requirement, as previously discussed, is to check the number
of input arguments before using them to avoid referencing a non existent
argument. However, it some case this might not be sufficient as the
underlying code imposes further constraints. For example an external
function call might be undefined if the input arguments are not
integers, or if one of the arguments is zero. Therefore, oct-files often
need additional input parameter checking.

There are several functions within Octave that might be useful for the
purposes of parameter checking. These include the methods of the
octave\_value class like `is_real_matrix`, etc., but equally include
more specialized functions. Some of the more common ones are
demonstrated in the following example

```
```
     #include <octave/oct.h>
     
     DEFUN_DLD (paramdemo, args, nargout, 
     	   "Parameter Check Demo.")
     {
       int nargin = args.length ();
       octave_value retval;
     
       if (nargin != 1)
         print_usage();
       else if (nargout != 0)
         error ("paramdemo: function has no output arguments");
       else
         {
           NDArray m = args(0).array_value();
           double min_val = -10.0;
           double max_val = 10.0;
           octave_stdout << "Properties of input array:\n";
           if (m.any_element_is_negative ())
             octave_stdout << "  includes negative values\n";
           if (m.any_element_is_inf_or_nan())
             octave_stdout << "  includes Inf or NaN values\n";
           if (m.any_element_not_one_or_zero())
             octave_stdout << 
     	  "  includes other values than 1 and 0\n";
           if (m.all_elements_are_int_or_inf_or_nan())
             octave_stdout << 
     	  "  includes only int, Inf or NaN values\n";
           if (m.all_integers (min_val, max_val))
             octave_stdout << 
     	  "  includes only integers in [-10,10]\n";
         }
       return retval;
     }
```
```

and an example of its use is

```
     paramdemo ([1, 2, NaN, Inf])
     => Properties of input array:
           includes Inf or NaN values
           includes other values than 1 and 0
           includes only int, Inf or NaN values
```
